# Short-term effect of intravitreal brolucizumab injections in patients with neovascular age-related macular degeneration on retinal nerve fiber layer thickness

**DOI:** 10.1038/s41598-023-32024-6

**Published:** 2023-04-24

**Authors:** Sung Yeon Jun, Daniel Duck-Jin Hwang

**Affiliations:** 1 Department of Ophthalmology, Hangil Eye Hospital, #35 Bupyeong-Daero, Bupyeong-Gu, Incheon, 21388 Korea; 2 Department of Ophthalmology, Catholic Kwandong University College of Medicine, Incheon, Korea

**Keywords:** Outcomes research, Macular degeneration

## Abstract

This study reported the short-term effects of intravitreal brolucizumab (IVB) on peripapillary retinal nerve fiber layer (RNFL) thickness in patients with neovascular age-related macular degeneration (nAMD). This retrospective observational case series included patients with nAMD treated with other anti-vascular endothelial growth factor (anti-VEGF) agents and subsequently switched to IVB because of poor response to those other agents on spectral domain optical coherence tomography (SD-OCT). Best-corrected visual acuity (BCVA), intraocular pressure, funduscopy, and SD-OCT were assessed at baseline, 2 weeks, 1 month, and 3 months after injection. Twenty-two patients were included in the study. In the IVB group, BCVA significantly improved 3 months after injection compared with baseline (0.45 ± 0.25 vs. 0.38 ± 0.25, *p* = 0.012). During the 3-month follow-up, compared with baseline, RNFL thicknesses of the global, superior temporal, inferior temporal, inferior nasal, nasal, and superior nasal sectors did not change substantially in the IVB group. However, temporal RNFL thickness significantly decreased at 1 month (*p* = 0.045), and the significance was lost at 3 months (*p* = 0.378). The central macular thickness of treated eyes significantly decreased compared with the baseline at every follow-up visit. IVB in patients with nAMD had morphological and functional visual gain effects without RNFL thinning during the short-term follow-up.

## Introduction

Age-related macular degeneration (AMD) is the primary cause of elderly blindness in industrialized countries^1^. With the development of anti-vascular endothelial growth factor (VEGF) medication, the visual outcome of patients with neovascular AMD (nAMD) has improved, and a meaningful reduction in the incidence of legal blindness has occurred^2,3^.

Brolucizumab is a single-chain humanized antibody fragment that was recently approved to treat nAMD^4^. The single-chain antibody fragment, which has a molecular weight of 26 kDa, has the potential for greater tissue penetration than early anti-VEGF therapies and enables the delivery of higher molar doses than larger molecules^5,6^. Additionally, brolucizumab was recently found to be non-inferior to aflibercept in two pivotal trials in terms of visual outcome^4^.

Although the efficacy of brolucizumab has been demonstrated, several issues, such as intraocular inflammation and retinal vasculitis, have been documented^7–11^. VEGF is a well-known neurotrophic factor found in the central nervous system, including the retinal nerve fiber layer (RNFL)^12–14^. In theory, suppression of neurotrophic cytokines could have negative consequences on the RNFL. Additionally, intraocular pressure (IOP) fluctuations caused by intravitreal injections can damage the RNFL^15–17^.

Anti-VEGF has been reported to change RNFL thickness, according to several reports. However, no studies have investigated the effect of brolucizumab injection on RNFL thickness. The aim of this single-center study was to report the real-world experience of intravitreal brolucizumab (IVB) injection and the effect of nAMD treatment on peripapillary RNFL thickness measured by spectral domain optical coherence tomography (SD-OCT).

## Results

### Baseline characteristics

Table 1 provides the patients’ demographics. Among the 22 patients, 19 were male (86.36%), and the mean age was 67.77 ± 5.95 years. The typical type of choroidal neovascularization was the most frequent (14 eyes, 63.6%), followed by polypoidal choroidal vasculopathy (PCV; 8 eyes, 35.4%), and there was no retinal angiomatous proliferation (RAP) type. None of the patients in the IVB group had undergone intravitreal injections other than aflibercept, bevacizumab, and ranibizumab, and the average number of previous injections was 16.50 ± 9.81 (range, 3–37). There were no significant differences in baseline characteristics, including IOP and peripapillary RNFL thickness, between the treated and fellow eyes.

**Table 1:** Baseline characteristics of all eyes that received intravitreal brolucizumab injection and untreated fellow eyes.

	IVB	Fellow eye	*p*-value
No. of patients	22	19	
Age (years)	67.77 ± 5.95	67.63 ± 4.08	0.927*
Sex (male/female)	19/3	16/3	0.848^†^
Normal/nAMD (n)	0/22	19/0	
Types of CNV			
PCV (n)	8	0	
Typical AMD(n)	14	0	
RAP (n)	0	0	
Systemic disease			
Hypertension (n)	14	12	0.975^†^
Diabetes (n)	2	2	0.879^†^
Treated eye laterality (OD/OS)	11/11	10/9	
No. of previous anti-VEGF injections (range)	16.50 ± 9.81 (3, 37)	None	
Corrected visual acuity (logMAR)	0.45 ± 0.25	0.14 ± 0.29	< 0.001*
Refractive error (SE)	− 0.01 ± 1.30	− 0.00 ± 1.11	0.979*
IOP (mmHg)	14.72 ± 3.45	13.68 ± 2.64	0.330*

*p value derived from Mann–Whitney *U*-test. ^†^*p* value derived from Pearson’s chi-square test; IVB, group that received intravitreal brolucizumab injection; nAMD, neovascular age-related macular degeneration; n, number; CNV, choroidal neovascularization; PCV, polypoidal choroidal vasculopathy; AMD, age-related macular degeneration; RAP, retinal angiomatous proliferation; OD, right eye; OS, left eye; VEGF, vascular endothelial growth factor; logMAR, logarithm of the minimum angle of resolution; SE, spherical equivalent; IOP, intraocular pressure.

### Changes in BCVA and IOP

**Figure 1:**
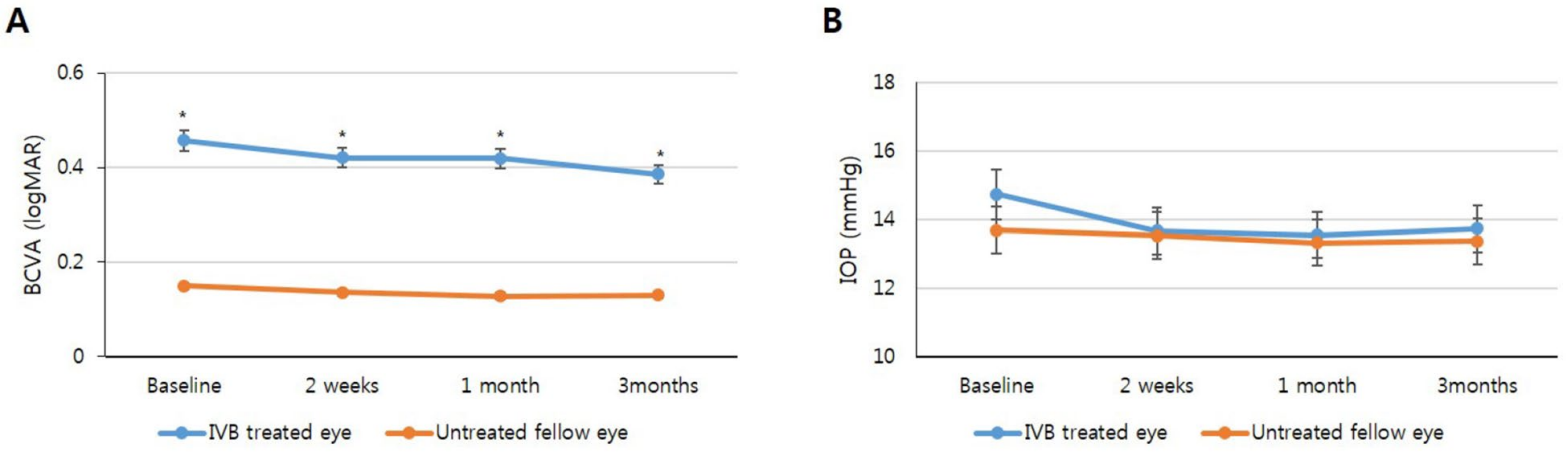
Changes in the best corrected visual acuity (BCVA) and intraocular pressure (IOP). (**A**) BCVA in the intravitreal brolucizumab (IVB) treated and untreated fellow eye groups. (**B**) IOP in the IVB treated and untreated fellow eye groups. The differences between the treated and control eyes are analyzed using the Mann–Whitney *U*-test, and significant *p* values are represented with asterisks.

In the IVB group, the logarithm of the minimum angle of resolution (logMAR) of the BCVA of the treated eyes significantly improved at 3 months (0.38 ± 0.25) after injection compared with the baseline value (0.45 ± 0.25, *p* = 0.012). There were no significant changes in visual acuity during the follow-up period in the fellow eye group. At all visits, the BCVA of the fellow eye was significantly higher than that of the IVB group (Fig. 1, *p* < 0.001).

In the treated eyes of the IVB group, the mean IOP before injection was 14.72 ± 3.45, and the mean IOP 1 day after injection was 13.77 ± 3.66. One day after the injection, there was no significant change in IOP (*p* = 0.070). Three months after injection, the IVB group experienced a borderline IOP reduction effect (13.72 ± 3.57 mmHg) compared to the baseline IOP (14.72 ± 3.45 mmHg), although the difference was nonsignificant (*p* = 0.093). In the eyes previously treated during the early phase with other anti-VEGF agents, there was no significant change in IOP compared to the baseline (*p* = 0.403). In the fellow eye group, there was no significant change in IOP compared to the baseline, and there was no significant difference in IOP compared to the IVB group at any visit time (Fig. 1).

### Change in the peripapillary RNFL thickness

The thicknesses of all RNFL sectors were not different between the IVB-treated eye, previously treated eyes during the early phase with other anti-VEGF agents, and fellow eyes at any visit (Table 2). However, compared to the baseline, the temporal RNFL thickness in the IVB-treated eyes had significantly decreased after 1 month (*p* = 0.045). However, the significance was lost (*p* = 0.375), and the RNFL thickness recovered to baseline at 3 months in the IVB-treated eyes. No other sectors showed any significant changes (Table 2). During the follow-up period, no significant change in RNFL thickness was observed in the fellow eyes and previously treated eyes with other anti-VEGF agents. No eyes were newly diagnosed with glaucoma during the study period.

**Table 2:** Longitudinal changes of the retinal nerve fiber layer thickness (μm) in the group administered intravitreal brolucizumab injection and the untreated fellow eyes.

	Treated eye with brolucizumab	Previously treated eye with other anti-VEGF agents	Fellow eye	*p*-value*	*p*-value**
Global sector RNFL					
Baseline	99.47 ± 14.19	100.42 ± 16.94	97.22 ± 12.19	0.849	0.460
1 month	98.18 ± 13.43	100.26 ± 16.67	96.94 ± 12.60	0.661	0.563
3 months	98.86 ± 14.73	100.15 ± 16.41	96.94 ± 12.65	0.792	0.581
*p*^†^ (base vs. 1 M)	0.286	0.857	0.929		
*p*^†^ (base vs. 3 Ms)	0.199	0.784	0.875		
*p*^†^ (1 M vs. 3 Ms)	0.598	0.542	1.000		
Superior temporal sector RNFL					
Baseline	126.33 ± 18.86	131.78 ± 24.24	126.38 ± 28.19	0.430	0.460
1 month	127.09 ± 20.75	132.05 ± 23.45	125.88 ± 28.72	0.477	0.657
3 months	130.45 ± 17.96	132.05 ± 23.09	126.11 ± 29.00	0.805	0.904
*p*^†^ (base vs. 1 M)	0.965	0.877	0.548		
*p*^†^ (base vs. 3 Ms)	0.930	0.876	0.972		
*p*^†^ (1 M vs. 3 Ms)	0.439	1.000	0.414		
Temporal sector RNFL					
Baseline	75.71 ± 9.78	74.47 ± 9.74	73.94 ± 12.00	0.690	0.646
1 month	70.77 ± 10.84	75.42 ± 9.72	72.55 ± 10.77	0.159	0.757
3 months	75.54 ± 17.98	75.57 ± 10.17	72.61 ± 10.82	0.994	0.778
*p*^†^ (base vs. 1 M)	0.045**	0.512	0.227		
*p*^†^ (base vs. 3 Ms)	0.378	0.443	0.197		
*p*^†^ (1 M vs. 3 Ms)	0.012**	0.625	0.655		
Inferior temporal sector RNFL					
Baseline	140.23 ± 31.40	142.00 ± 28.81	141.77 ± 29.34	0.855	0.945
1 month	139.04 ± 28.86	143.21 ± 35.58	143.66 ± 30.98	0.681	0.427
3 months	139.77 ± 29.71	143.47 ± 35.68	143.50 ± 30.93	0.719	0.545
*p*^†^ (base vs. 1 M)	0.797	0.544	0.676		
*p*^†^ (base vs. 3 Ms)	0.732	0.461	0.864		
*p*^†^ (1 M vs. 3 Ms)	0.502	0.384	0.180		
Inferior nasal sector RNFL					
Baseline	116.76 ± 27.90	115.26 ± 24.81	113.83 ± 20.35	0.859	0.666
1 month	116.77 ± 26.04	114.63 ± 25.82	113.27 ± 20.84	0.794	0.946
3 months	114.72 ± 24.64	114.52 ± 25.62	112.88 ± 20.90	0.980	0.861
*p*^†^ (base vs. 1 M)	0.723	0.594	0.972		
*p*^†^ (base vs. 3 Ms)	0.469	0.518	0.529		
*p*^†^ (1 M vs. 3 Ms)	0.246	0.790	0.180		
Nasal sector RNFL					
Baseline	71.76 ± 14.20	76.10 ± 26.02	71.77 ± 13.13	0.511	0.900
1 month	74.09 ± 17.57	74.68 ± 21.87	71.50 ± 13.00	0.924	0.968
3 months	70.18 ± 15.61	73.26 ± 17.16	71.33 ± 12.95	0.551	0.581
*p*^†^ (base vs. 1 M)	0.936	0.260	0.904		
*p*^†^ (base vs. 3 Ms)	0.234	0.339	0.647		
*p*^†^ (1 M vs. 3 Ms)	0.306	0.448	0.180		
Superior nasal sector RNFL					
Baseline	117.14 ± 28.10	109.27 ± 21.64	105.55 ± 17.76	0.165	0.174
1 month	115.31 ± 27.63	111.26 ± 22.91	105.72 ± 20.09	0.615	0.325
3 months	114.90 ± 28.22	113.00 ± 28.83	105.94 ± 19.91	0.659	0.199
*p*^†^ (base vs. 1 M)	0.147	0.894	0.176		
*p*^†^ (base vs. 3 Ms)	0.631	0.561	0.346		
*p*^†^ (1 M vs. 3 Ms)	0.721	0.495	0.317		

The significant *p*-values are shown in [bold]. *Comparison between treated eyes with brolucizumab and previously treated eyes during the early phase with other anti-vascular endothelial growth factor agents (Mann–Whitney *U*-test). **Comparison between treated eyes with brolucizumab and untreated fellow eyes in each period (Mann–Whitney *U*-test). ^†^Comparison between the baseline and follow-up visits for each value (Wilcoxon signed-rank test). RNFL, retinal nerve fiber layer; base, baseline; M, month.

### Central macular thickness

At baseline, the central macular thickness (CMT) of the IVB-treated eyes was significantly higher than that of the fellow eyes (Table 3). When compared to the baseline, the CMT of the IVB group was significantly decreased after 2 weeks, 1 month, and 3 months. However, at 3 months after IVB injection, a significant increase was observed in the CMT compared to 1 month after injection (*p* = 0.007). The longitudinal changes of CMT in the fellow and previously treated eyes during the early phase with other anti-VEGF agents were not significant (Table 3).

**Table 3:** Central macular thickness (μm) before and after intravitreal brolucizumab injection.

	Treated eyewith brolucizumab	Previously treated eye with other anti-VEGF agents	Fellow eye	*p-*value*	*p-*value**
Baseline	452.40 ± 174.19	407.42 ± 162.04	302.89 ± 70.59	0.400	< 0.001
1 month	294.13 ± 86.66	394.68 ± 166.01	299.89 ± 62.54	0.194	0.565
3 months	328.40 ± 106.84 84	380.63 ± 145.68	301.15 ± 61.47		0.629
*p*^†^ (base vs. 1 M)	< 0.001	0.474	0.513		
*p*^†^ (base vs. 3 Ms)	< 0.001	0.359	0.842		
*p*^†^ (1 M vs. 3 Ms)	0.007	0.583	1.000		

*Comparison between treated eyes with brolucizumab and previously treated eyes during the early phase with other anti-vascular endothelial growth factor (VEGF) agents (Mann–Whitney *U*-test). **Comparison between treated eyes with brolucizumab and untreated fellow eyes in each period (Mann–Whitney *U*-test). ^†^Comparison values between the baseline and follow-up visits for each value (Wilcoxon signed-rank test). M, month; base, baseline.

## Discussion

Here, we report the single-center clinical use of brolucizumab. Our findings indicate that IVB therapy in patients with nAMD who were previously treated with multiple anti-VEGF agents without satisfactory resolution of fluid compartments had morphological and functional visual gain effects without RNFL thinning during the short-term follow-up period.

Studies have shown that the IOP could drastically increase from baseline after intravitreal injection^15,16^. Additionally, as VEGF plays a role in neuronal cell survival and has neuroprotective, neurotropic, and angiogenic effects, anti-VEGF therapy may also disrupt VEGF’s neurophysiologic function, which may damage the optic nerves^12–14^. Martinez-de-la-Casa et al.^18^. observed a considerable decrease in the average RNFL thickness 12 months after ranibizumab injection in AMD patients. They reported that RNFL thinning after repeated intravitreal anti-VEGF injections might be related to drug toxicity or IOP fluctuation^18^. Wang et al.^19^ also reported dose response with RNFL thinning after multiple injections. Other studies^20–24^ found no significant differences in RNFL thickness after bevacizumab^20–22^ or ranibizumab^20–24^ injections. Shin et al.^22^ reported the meta-analysis findings that IOP fluctuation and the number of anti-VEGF injections had no adverse effects on RNFL thickness. Shah et al.^25^ also found no relationship between glaucoma development and injections but found that patients with glaucoma receiving injections required more topical anti-glaucoma therapies. Dail et al.^26^ found no difference between the other three available anti-VEGF agents (aflibercept, ranibizumab, and bevacizumab) in terms of RNFL thinning rate but found that thinning occurred over 2 years of follow-up. According to Ahn et al.^27^, multiple injections of ranibizumab and aflibercept did not cause a significant difference in peripapillary RNFL thickness in treatment-naïve patients with nAMD compared with the contralateral normal eye.

Brolucizumab has recently been approved as a new anti-VEGF agent for intravitreal administration in patients with nAMD. Only a few reports of its clinical outcomes in real-world settings are available to date. There have been some reports about the clinical outcomes, including BCVA, CMT, and retinal fluid compartments after brolucizumab injection in patients with nAMD^4,28–30^. However, to the best of our knowledge, this is the first study to evaluate changes in peripapillary RNFL thickness after brolucizumab injection in patients with intractable nAMD. In the present study, there was no IOP increase 1 day after the IVB injection. Additionally, the global RNFL thickness remained unchanged during the 3 months follow-up period after the IVB injection. However, the temporal RNFL thickness significantly decreased 1 month after IVB injection, but the significance was lost 3 months after IVB injection with an increasing tendency of macular edema. Longitudinal changes in RNFL thickness were not significant in any quadrant except the temporal area after injection in the present study. We do not think that the observed reduction in the temporal RNFL thickness of the treated eyes at 1 month after IVB injection was an adverse effect of anti-VEGF injection because the global and other five sectors of RNFL thickness did not show a significant reduction. This reduction may instead be related to the temporal anatomical improvement of macular exudative lesions 1 month after the IVB injection. Longer-term studies on the effect of IVB on RNFL thickness with more participants are needed in the future. In addition, it seems necessary to consider that the effect of IVB on naive and non-naive eyes may differ.

The reduction in temporal RNFL thickness in the pathological area is more likely due to a change in the macular lesion rather than a result of increased pressure or neurotoxicity due to anti-VEGF administration. That is, RNFL thickness may have increased at baseline due to an exudative lesion, edema, or hemorrhage in the macular area, and RNFL thickness was reduced 1 month after injection because the exudative lesion was depressed after the injection. Ahn et al.^27^ described changes in the macular tomography as a reason for decreased temporal RNFL thickness to the borderline after intravitreal aflibercept or ranibizumab injection. Therefore, a similar decrease in temporal sector RNFL thickness following IVB injection in patients with nAMD may be related to changes in macular tomography as a result of macular edema improvement.

This study had some limitations. The first limitation of this study was its retrospective design and the relatively small number of patients enrolled. Second, there was no sub-analysis of the different effects of brolucizumab according to the nAMD subtypes, including PCV and RAP and the prior numbers of intravitreal injection. Since brolucizumab is a new drug that has been commercialized in Korea, there was a limited number of target patients. Finally, the follow-up period was relatively short, with a total of 3 months. Despite these limitations, this study provides valuable data regarding the effect of brolucizumab injection on peripapillary RNFL thickness in non-naïve patients with nAMD.

In summary, no significant difference in global peripapillary RNFL thickness was observed in the treated eyes during the follow-up period after IVB injection. The RNFL thickness of the temporal sectors significantly decreased only 1 month after injection, presumably owing to the anatomical improvement of macular lesions. Therefore, a single intravitreal brolucizumab injection seems to be safe in terms of peripapillary RNFL damage in patients with intractable nAMD. We believe that this study can help alleviate the concerns of physicians regarding RNFL damage after IVB injection.

## Methods

### Participants

This was a retrospective, observational, and consecutive case series study. Patients diagnosed with nAMD at our hospital between April 2021 and March 2022 were included. A single ophthalmologist (DDH) delivered IVB (Beovu, Novartis AG, Basel, Switzerland and Genentech, Inc., South San Francisco, CA, USA) injections to 22 patients. All patients in the IVB group (6 mg/0.05 mL) had previously received anti-VEGF therapy, including bevacizumab, ranibizumab, and/or aflibercept, for nAMD, and those who continued to show fluid accumulation on SD-OCT with poor response were included. The eyes in the early phase previously treated with other anti-VEGF agents before brolucizumab injection were set as the control group 1. The normal contralateral eyes were considered as the control group 2, whereas contralateral eyes diagnosed with nAMD were excluded from the control group 2. PCV was diagnosed based on the presence of polypoidal lesions, with or without branching vascular networks. Cases that exhibited retinal-retinal or retinal-choroidal anastomoses were classified as type 3 neovascularization (RAP) ^31,32^. The remaining patients not diagnosed with either PCV or RAP were classified as having typical nAMD with type 1 or 2 choroidal neovascularization^33^. Medical records and SD-OCT (Spectralis OCT, Heidelberg Engineering, Heidelberg, Germany) data at baseline, 2 weeks, 1 month, and 3 months after injection were retrospectively reviewed. The exclusion criteria were as follows: (1) other ophthalmic diseases, such as retinal vascular disease, uveitis, glaucoma, and optic nerve disease. Patients with glaucoma were diagnosed using the Cirrus high-definition OCT (Carl Zeiss Meditec, Dublin, CA) and standard automated perimetry (SAP) using the 24-2 Swedish Interactive Threshold Algorithm standard automated visual field test (Humphrey Visual Field Analyzer; Carl Zeiss Meditec) before being enrolled in the study. They were defined as those with a glaucomatous disc appearance based on stereo disc photography corresponding to repeatable VF loss on two consecutive SAP evaluations. The glaucomatous disc appearance was defined as either a diffuse or localized neuroretinal rim thinning, notching, excavation associated or not with peripapillary atrophy, and optic disc hemorrhages^34^; (2) intraocular inflammation after IVB injection; and (3) peripapillary RNFL thickness not measured during the follow-up period.

### Ophthalmic examinations

At baseline, all patients underwent fluorescein angiography and indocyanine green angiography. One day after the injection, IOP measurement and slit-lamp microscopy were performed. At every 2-week, 1-month, and 3-month visit, binocular BCVA and IOP were assessed using slit-lamp biomicroscopy, fundus photography, and SD-OCT. The SD-OCT program (Spectralis Nsite Axonal Analytics Software; Heidelberg Engineering) measured the peripapillary RNFL thickness. Temporal (315–45°), superior temporal (45–90°), superior nasal (90–135°), nasal (135–225°), inferior nasal (225–270°), and inferior temporal (270–315°) RNFL thicknesses were assessed. The total 360° peripapillary RNFL thickness readings were averaged to obtain the global RNFL thickness. The study excluded tests in which the RNFL OCT quality did not meet the automatic real-time score of ≥ 16 and had a signal-to-noise ratio of ≥ 15 dB. A volume scan of 30° centered on the fovea with a central fixation assist and a 250-μm distance between scans was used to determine macular thickness. The average thickness of the center 1-mm-diameter circle was measured to confirm the CMT.

### Ethics statement

This study was conducted in accordance with the principles of the Declaration of Helsinki. The Institutional Review Board (IRB) of Hangil Eye Hospital (IRB number: 22004) approved this study and waived the requirement for informed consent from the study participants due to the retrospective nature of the study.

### Statistical analysis

SPSS Statistics 23 was used to conduct the statistical analysis (SPSS Inc., Chicago, IL, USA). Data are expressed as mean ± standard deviation. The Mann–Whitney *U*-test was used to compare the IVB-treated eyes to the control eyes. The Wilcoxon signed-rank test was used to examine changes in values from the baseline assessment to the 3-month follow-up. Statistical significance was considered when the *p*-value was < 0.05.

## Data Availability

On reasonable request, the corresponding author will provide the datasets created and analyzed during the current work.
